# Molecular genetics research in sub-Saharan Africa: how can the international community help?

**DOI:** 10.1186/s11568-014-0002-2

**Published:** 2014-09-17

**Authors:** Endashaw Bekele, Walter F Bodmer, Neil Bradman, Ian W Craig, Julie Makani, Sue Povey, Charles Rotimi

**Affiliations:** 1 College of Natural Sciences, Addis Ababa University, P.O. Box 3434, Addis Ababa, Ethiopia; 2 Department of Oncology and The Weatherall Institute of Molecular Medicine, University of Oxford, John Radcliffe Hospital, Oxford, OX3 9DS UK; 3 Henry Stewart Group, Russell House, 28/30 Little Russell Street, London, WC1A 2HN UK; 4 MRC SGDP Centre, Institute of Psychiatry, King’s College London, PO Box 82, London, SE5 8AF UK; 5 Department of Haematology and Blood Transfusion, Muhimbili University of Health and Allied Sciences, Box 65001, Dar-es-Salaam, Tanzania; 6 Nuffield Department of Clinical Medicine, University of Oxford, Oxford, UK; 7 Department of Genetics, Evolution and Environment, Darwin Building, UCL, Gower Street, London, WC1E 6BT UK; 8 Center for Research on Genomics and Global Health, National Human Genome Research Institute, National Institutes of Health, Building 12A, Room 4047, 12 South Drive, MSC 5635, Bethesda, MD 20892 USA

## Abstract

**Background:**

Opportunities provided by rapidly increasing access to educational resources, clinical and epidemiological data, DNA collections, cheaper technology and financial investment, suggest that researchers in sub-Saharan Africa outside South Africa (SSAOSA) could now join the genomics revolution on equal terms with those in the West.

**Findings:**

Current evidence, however, suggests that, in some cases, various factors may be compromising this development. One interpretation is that urgent practical problems, which may compromise motivation, aspiration and ambition, are blocking opportunity.

**Conclusions:**

Those wishing to help should support the SSAOSA scientists both at the level of extending collaboration networks and in stimulating academic leadership at national and institutional levels to ensure adequate resources are allocated. Members of organisations representing the international community of human geneticists, such as HUGO, have a significant responsibility in supporting such activities.

Scientists in sub-Saharan Africa have, over the next few years, a real opportunity to break through and join their colleagues on the world stage in pushing forward the frontiers of research in genomics. A number of separate elements have now come together to make this possible. They have that most important resource on their doorstep – human genetic diversity. Africa, the birthplace of anatomically modern man, has it in abundance. Furthermore, the costs of genotyping and sequencing DNA have been falling rapidly. Attention has turned increasingly to the importance of data, which includes creating good biobanks of DNA and associated medical and other records as a resource for biomedical data for genotype/phenotype analysis. Most African countries have had some form of investment in improving medical records in health facilities as well as population-based data such as census information. These activities have been enabled now by cheaper technology including mobile phone and web-based tools. However, they are nevertheless labour intensive. This can give African laboratories a cost advantage if they have the expertise and this expertise is harnessed by national and international funding agencies. So has a new day dawned? Is there now the environment that African researchers including geneticists, epidemiologists and clinicians have waited for? For some the answer may be yes. But for some, specifically geneticists in SSAOSA (sub-Saharan Africa outside South Africa), the answer is less certain. In attempting to answer this question, we evaluate three opportunities to enable active genomic research in Africa; access to knowledge through publications, adequate and sustainable funding and a critical mass of biomedical scientists in the field of genomics.

## Findings

Acquiring up to date knowledge has always been a barrier to undertaking genetic research in SSAOSA. However, even here, there is good news. As a report by The Association of Commonwealth Universities (ACU - http://www.acu.ac.uk/growing knowledge) shows, this is no longer the problem it once was. Many, possibly all, universities in SSAOSA which are likely to have molecular geneticists on their staff should now have almost the same access to journals and ebooks as their counterparts in the North. The International Network for the Availability of Scientific Publications (INASP – http://www.inasp.info/) has done a superb job in securing this outcome. This transformation, combined with continuously improving internet access, means that very shortly the latest lectures, webinars and seminars accessed by the leading universities in the most developed countries will be available in real time. The recent initiative by Coursera (https://coursera.org/) in which entire courses from leading universities are being made available online completely free is one example of easily accessible educational resources. Another is provided by the collaboration between the Human Genome Organisation (HUGO) (http://www.hugo-international.org/index.php) and Henry Stewart Talks through which a collection of over 1,500 lectures on biomedicine and the life sciences by world leading experts including Nobel Laureates (http://hstalks.com/) is being provided either free or at a massive discount to SSAOSA universities. Unfortunately, there appear to be factors that hinder scientists in Africa accessing this freely available knowledge. To understand why this may be so, it is worth returning to the study by the ACU. Undertaking an in depth appraisal of four SSAOSA universities in Kenya, Malawi, Rwanda and Tanzania it concluded *“The problem of availability – that is the provision of affordable or free journals and other resources in online form – has been widely and successfully addressed over several years”*. However the study also reports that *“Awareness of the materials available amongst staff and students is low”*. One interpretation that reconciles these two observations is that other factors and urgent practical problems, which may compromise motivation, aspiration and ambition, are blocking opportunity. As all researchers know, however, access to samples, equipment and knowledge is usually not enough. Money is often the most important consumable. Here too there is good news in the launch of the Human Heredity and Health in Africans (H3Africa) programme funded by the UK’s Wellcome Trust and the US’s NIH (working with the African Society of Human Genetics (AfSHG)). Over the next five years they will, together, be awarding grants in excess of 70 million US dollars directly to African principal investigators based on the continent. This will in turn lead to increases in appropriately trained manpower which can promote both national and international collaborations. It is hoped that the launch of H3Africa will lead to an increase in funding by government and other organisations. This is critical as it is recognised that the initial investment by H3Africa will not be adequate to ensure the required growth of genomics in Africa. So far, the more than 70 million US dollars for H3Africa has funded several projects including disease-specific studies, one Pan-African bioinformatics Network and two pilot biobanking projects. MalariaGEN, a consortium looking at genetic determinants of malaria, was established in 2005 with funding of 16.4 million dollars from the Wellcome Trust and Gates foundation through the National Institutes of Health.

The third element is human resource. To gain quickly some insight into the number of researchers active in human genetics at a senior level native to and currently working in SSAOSS, we did four things. Firstly we looked at the AfSHG website which listed the members of both the Association’s Executive Committee and the Scientific Programme Committee for the joint AfSHG/South African Society of Human Genetics 2011 meeting (http://www.afshg.org). Not one of the ten members of the Executive Committee nor of the 19 members of the Scientific Programme Committee had an affiliation solely to an institution in SSAOSA. Next we looked at the Wellcome Trust’s H3Africa Applicant Discussion Group website (http://www.nature.com/scitable/groups/h3africa-applicant-discussion-group-22066126) which is described as a *“discussion and collaborative space for prospective applicants”*. There were 62 individuals registered on 8 August 2013 of which 16 were from SSAOSA. Of these, nine had first or senior author positions in publications listed in a PubMed search. We then undertook PubMed searches selecting as filters four countries, two in the west (Nigeria and Cameroon) and two in the east (Ethiopia and Kenya) to identify publications on some aspect of human molecular genetics or the molecular genetics of pathogens of humans that appeared in 2012 with a SSAOSA country included in the Authors’ address. In Nigeria we identified 17, in Cameroon 6, in Ethiopia 5 and in Kenya 15. Finally, we contacted the Wellcome Trust, NIH and AfSHG to gain information on the numbers of researchers primarily resident in SSAOSA active in human genetic research. While the results of our enquiries are partly anecdotal, it is clear that there is a small but active group of SSAOSA geneticists undertaking research sufficiently well to get published in internationally recognised peer reviewed journals.

What can already established scientists in South Africa and elsewhere do to help SSAOSA researchers and extend the numbers of those who may wish to become involved? The first action is to support sustainable partnerships which ensure equitable involvement by all partners. The second is to work with institutions and funders to increase efforts to stem the loss, by emigration, of potential African field leaders and encourage ‘brain circulation’ rather than ‘brain drain’. Researchers in the North should be open to collaborations where the ‘lead scientist’ is from Africa. After all, as observed at the start of this article, SSAOSA researchers are interested in, and have opportunities to work on, questions that are of global interest. Established scientists must not crowd out emerging scientists from SSAOSA. If SSAOSA research is to be sustainable it has to be locally motivated and locally driven; but the necessary levels of support must be in place. As we have noted, the apparently available opportunity seems difficult to access locally; a situation that may require addressing at the political level. In short, moves to correct this mismatch will have to come from the academic leadership at national and institutional levels and from the staff and students themselves. Those wishing to help should support the SSAOSA scientists in these endeavours so that they may create the momentum, lobby their governments and ensure adequate resources are allocated. With the right environment SSAOSA researchers have, for the first time, the opportunity in many areas of human molecular genetics to contribute on playing fields that are becoming more level. Members of organisations representing the international community of human geneticists, such as HUGO, have a significant responsibility in supporting such opportunity.

